# Healthcare interventions for the prevention and control of gestational diabetes mellitus in China: a scoping review

**DOI:** 10.1186/s12884-017-1353-1

**Published:** 2017-06-05

**Authors:** Tingting Xu, Yasheng He, Livia Dainelli, Kai Yu, Patrick Detzel, Irma Silva-Zolezzi, Sheri Volger, Hai Fang

**Affiliations:** 1 China Center for Health Development Studies, Peking University, Haidian District, PO Box 505, Beijing, 100191 China; 2 Department of Health Policy and Management, School of Public Health, Peking University, Beijing, China; 3 Nestlé Research Center, Lausanne, Switzerland; 4 Nestlé Research Center, Beijing, China; 5 Nestlé Nutrition, King of Prussia, PA USA

**Keywords:** Gestational diabetes mellitus (GDM), China, Healthcare, Interventions, Prevention, Glycemic control

## Abstract

**Background:**

Gestational Diabetes Mellitus (GDM) is a type of diabetes which occurs during pregnancy. Women with GDM are at greater risk of complications during pregnancy and delivery, while babies born from mothers with GDM are at greater risk of post-natal complications. Using the most updated diagnosis criteria, the GDM prevalence is estimated at 9.3–25.5% worldwide and 9.3–18.9% in China. Our objective was to identify healthcare interventions aimed at GDM prevention and control in China.

**Methods:**

A best-evidence synthesis was performed based on a systematic search of literature published between 1997 and October 2015 in PubMed, Web of Science, China National Knowledge Infrastructure (CNKI), and Wan-fang databases using keywords “Gestational Diabetes Mellitus”, “GDM”, “Intervention” “Medical Intervention” “Early Medical Intervention”, “Dietary Intervention”, “Exercise Intervention”, “Lifestyle Intervention”, “Therapy”, “Treatment” and “China”. Inclusion criteria were studies conducted in China, reporting GDM healthcare interventions, and published in either Chinese or English. Two reviewers independently assessed eligibility and quality of the studies and extracted the data. Treatment efficacy was examined with weighted pooled odds ratio (OR) meta-analyses.

**Results:**

The search resulted in 5961 articles (published in 276 different Chinese language journals and 6 English language journals), of which 802 were included in this synthesis. While 39.4% (*n* = 316) failed to report the GDM diagnostic criteria used, the remaining studies classified GDM with various international (*n* = 5) or Chinese (*n* = 7) diagnostic standards. Treatment interventions were categorized into 6 types: dietary (18.6%), exercise (1.6%), medication (20.7%), health education (9.0%), psychological (2.6%) and combination (47.4%). No interventions aimed at GDM prevention were identified. Meta-analyses demonstrated a statistically significant overall benefit of GDM treatment strategies in reducing the odds of maternal and infant adverse outcomes (ORs range 0.20–0.34, 95% CI 0.17–0.49, *P* < 0.05 for all). Dietary, western medication, and combined interventions were the most effective inteventions.

**Conclusions:**

An increasing number of healthcare interventions were found in China aimed at controlling GDM while no interventions were intended for GDM prevention. Well-designed clinical trials are needed to determine the comparative and cost effectiveness of GDM prevention and treatment strategies.

**Electronic supplementary material:**

The online version of this article (doi:10.1186/s12884-017-1353-1) contains supplementary material, which is available to authorized users.

## Background

Every pregnancy is associated with a certain degree of hyperinsulinemia and insulin resistance, but in some women these physiological changes lead to Gestational Diabetes Mellitus (GDM) [1]. GDM is a condition in which women without previously diagnosed glucose intolerance exhibit high blood glucose levels during pregnancy, particularly during their third trimester [2]. Over the past 20 years, the prevalence of GDM has increased world-wide, and across China [3]. According to the latest diagnostic criteria established by the International Association of Diabetes and Pregnancy Study Groups (IADPSG) in 2010 [4, 5] the GDM prevalence was estimated at 9.8–25.5% worldwide and 9.3–18.9%% in China [6, 7].

GDM has a significant impact on the health of both the mother and the baby. Women with GDM are at greater risk of pregnancy complications, such as pre-eclampsia, pre-term birth and macrosomia [8], as well as post-partum complications, such as a higher risk of GDM in subsequent pregnancies and the development of type II diabetes up to 25 years after the childbirth [9, 10]. Of greatest concern, however, are the profound epigenetic changes that GDM has, through the intrauterine environment, on the offspring, who display a increased risk of short- and long-term health effects. In the post-natal period, potential adverse outcomes include neonatal hypoglycemia, macrosomia, hyperbilirubinemia and respiratory distress syndrome [9]. In later life, babies born from mothers with GDM have a higher risk of type II diabetes, obesity, and metabolic syndrome [11–13]. In turn, these traits are transmitted to the next generation, further perpetuating the vicious cycle of metabolic diseases [14, 15].

Recent evidence indicates that GDM treatments can effectively improve some maternal and newborn health outcomes [16, 17]. In 2014, the Department of Obstetrics and Gynecology and the Department of Perinatal Medicine within the Chinese Medical Association developed a joint recommendation for the diagnosis and treatment of GDM in China [18]. The guideline is similar to the American Diabetes Association and International Association of Diabetes and Pregnancy Study Group (IADPSG) guideline [19, 20] and recommends that all non-diabetic women undergo diagnostic testing with a 75 g Oral Glucose Tolerance Test (OGTT) at the 24-28th weeks of pregnancy. GDM is defined as an OGTT fasting glucose level higher than 5.1 mmol/L (92 mg/dl), 1 h post-OGTT higher than 10.0 mmol/L (180 mg/dl), or 2 h post-OGTT higher than 8.5 mmol/L (153 mg/dl). Women diagnosed with GDM should receive lifestyle interventions (including intensive diet, exercise, and/or health education). Medication interventions (e.g., insulin therapy) may be recommended for women who do not respomnd to lifestyle interventions or with diabetic ketoacidosis. There are minor differences between China and Western guidelines in lifestyle treatment recommendations. For example, the exercise time and intensity recommended in China are lower than those in the United States, Canada, and Australia [18, 21].

Considering the rising rate of GDM-related morbidity and adverse outcomes in mothers and babies in China, there is an impending and great need for effective GDM treatment strategies along with GDM prevention programs. Despite a large number of GDM clinical trials conducted over past 20 years in China, there has been no systematic search of the literature describing the types of healthcare interventions targeting GDM and examining comparative effectiveness. Therefore, the objective of this review was to identify and provide a descriptive overview of healthcare interventions aimed at the prevention and control of GDM in China. The secondary objective was to examine the effectiveness of these treatment strategies in reducing the risk of adverse health outcomes.

## Methods

### Literature search

A best-evidence synthesis was performed based on a systematic search of literature published between 1997 and October 2015 conducted in PubMed, Web of Science, China National Knowledge Infrastructure (CNKI), and Wan-fang databases using the keywords “Gestational Diabetes Mellitus”, “GDM”, “Intervention” “Medical Intervention” “Early Medical Intervention”, “Dietary Intervention”, “Exercise Intervention”, “Lifestyle Intervention”, “Therapy”, “Treatment” and “China”. These keywords were used alone and in different combinations. All potentially eligible studies in either English or Chinese were considered for review.

### Study selection

Articles included in the analysis were selected according to the following criteria: 1) evaluating the effects of a healthcare interventions on GDM and reporting maternal and newborn health outcomes; 2) original research articles; 3) conducted in China; and 4) full journal publications. Meeting abstracts, letters to the editor, treatment guidelines or recommendations, expert opinions, and narrative reviews were excluded.

One researcher applied the search strategy across the four databases. All the retrieved records were then assigned to three independent researchers, who screened titles and abstracts according to the inclusion criteria. Finally, two reviewers independently reviewed the full text of the included articles, and one additional reviewer cross-checked that the articles included by the two reviewers met the inclusion criteria.

Further appraisal of full-text articles was based on the reporting of at least one key maternal and/or neonatal outcomes: hypertensive disorders, postpartum hemorrhage, premature rupture of membranes, postnatal infection, uterine-incision delivery, polyhydramnios, abortion, ketoacidosis, macrodome, preterm birth, neonatal hyperbilirubinemia, fetal distress, neonatal hypoglycemia, asphyxia neonatorum, dead fetus, and abnormal fetus. Any articles not reporting a key GDM health outcome were not considered a GDM healthcare intervention and thus excluded from the final analysis.

### Data extraction

According to the aforementioned criteria, 802 articles were included in this synthesis and their full texts were obtained. Data from the articles were extracted using the standardized data abstraction form recommended by Cochrane and the most relevant data were included in the descriptive analyses [22]. The extracted data was appraised for the following parameters: general article information (authors, publication year, title, journal, and language), study population characteristics (sample selection and maternal characteristics), study objectives (prevention or control treatment), classification by type of healthcare intervention (diet, exercise, medication, combined), treatment details and duration, study methods, and maternal and newborn outcomes. Data extraction was performed independently by two researchers. Any data discrepancy was resolved after reviewing the data source and upon discussion with the third researcher. Since the objective of this review was to report all the different types of current GDM healthcare interventions in China, frequency analyses were used to present the main findings. Finally, to evaluate the effectiveness of the interventions, weighted pooled odds ratio meta-analyses were conducted among the GDM interventions strategies reporting adverse maternal and/or infant health outcomes. All statistical analyses were conducted using SPSS version 18 and Stata 2014.

## Results

### Literature search

**Fig. 1:**
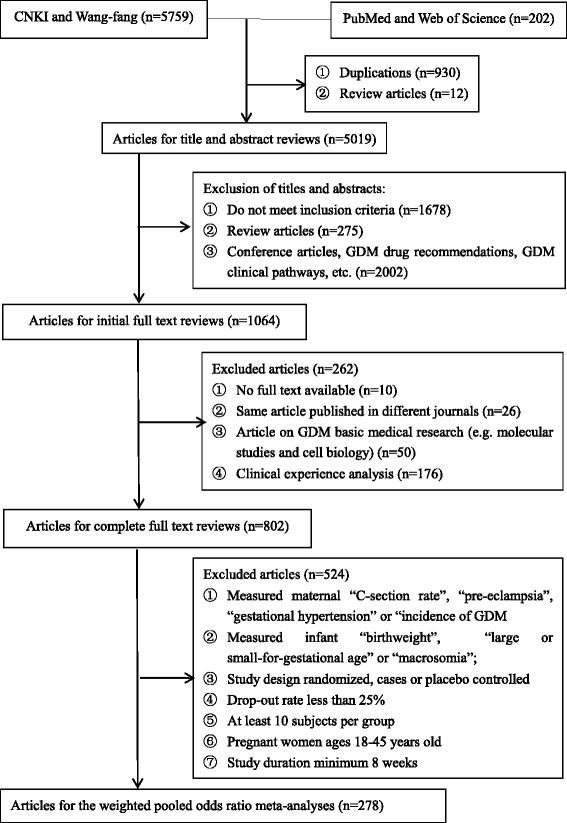
Flow diagram of the literature search and article screening

A total of 5961 articles from both Chinese (CNKI and Wan-fang) and English databases (PubMed and Web of Science) were identified. First, 940 duplicated articles and 12 review articles were excluded. Of the 5019 articles left for title and abstract screenings, excluded were 1678 for inappropriate study objectives (not related to GDM healthcare interventions); 275 were review articles, and 2002 did not meet the inclusion criteria for a variety of reasons (ie., conference synopses, GDM drug recommendations, and/or GDM clinical guidelines). The full texts of 1064 articles were reviewed for data extraction. Amongst these, 262 additional articles were excluded because: the full text was not available (*n* = 10), the same article was published in different journals (*n* = 26), the article was not a healthcare interventions [(i.e., GDM molecular studies and cell biology studies, (*n* = 50)], or they were articles on the analysis of a clinical experience (*n* = 176). In total, 802 articles, published in 276 different Chinese language journals and 6 English language journals, on GDM healthcare interventions in China were included in this synthesis for full text data extraction and further detailed analysis. The full search strategy is shown in Fig. 1.

### Study characteristics

**Fig. 2:**
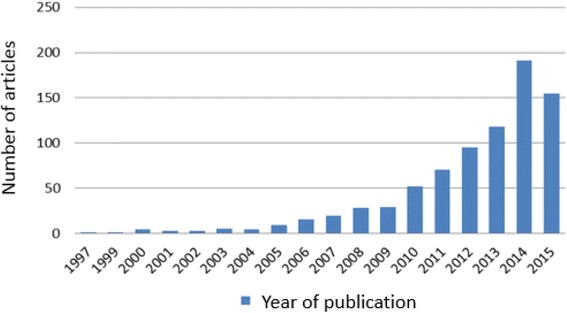
Number of articles published per year from January 1997 to October 2015

The first study on GDM healthcare interventions in China was published in 1997. The number of annual publications increased from 1 in 1997 to 52 in 2010, and to 191 in 2014 (Fig. 2).

In less than 20 years, more than 800 articles reporting GDM healthcare interventions in China were published in 276 Chinese journals (98%) and 6 English journals (2%). Among the Chinese journals, 43 (15%) were Chinese core journals, well recognized for their quality in China. The top 5 journals in which the articles were published, ordered by the numbers of publications (the journals’ Chinese names were here translated in English) were: Diabetes New World (68 articles), Guide of China Medicine (28 articles), Medical Information (28 articles), China Modern Medicine (16 articles), and China Practical Medicine (16 articles). The majority of first authors were affiliated with general hospitals (61%), with the rest associated with maternal and children’s hospitals and community health centers. The published studies were mainly from Eastern China, particularly from Shanghai (16%) and Guangzhou (5%).

### Types of interventions for GDM

We identified 6 types of GDM health interventions (Table 1) in China: dietary (*n* = 149, 18.6%), exercise (*n* = 13, 1.6%), medication (*n* = 166, 20.7%), health education (*n* = 72, 9.0%), psychological (*n* = 21, 2.6%) and combination (*n* = 381, 47.5%). The main first-line treatment strategy to control glucose levels was a combination of dietary and exercise [23]. Whenever first-line interventions were not effective, medication therapies, including Western medicine (*n* = 156, 19.5%), Chinese traditional medicine (*n* = 6, 0.7%) and integrated Chinese and Western medicine intervention (*n* = 4, 0.5%), were administered [24]. Health education interventions provided key information regarding GDM to pregnant women and promoted appropriate diet, exercise, and compliance with the prescribed medications [25]. Although exercise intervention could aid in reducing glucose levels providing exercise instructions to pregnant women [26], it was rarely identified as a stand-alone intervention but was most frequently implemented along with other types of GDM interventions. A high proportion of studies, used a combination of health interventions for GDM, due to the complexity of GDM and the strong influence of lifestyle factors in its pathology. There were three combined GDM health interventions: 1) dietary + exercise (hereafter called DE, *n* = 34, 4.2%); 2) dietary + exercise + medication (hereafter called DEM, *n* = 130, 16.2%); and 3) dietary + exercise + medication + health education + psychological (hereafter called DEMHP, *n* = 217, 27.1%). No interventions were intended for GDM prevention in China.

**Table 1:** Types of GDM health interventions used in China

Intervention	Number of studies	Percentage (%)
Dietary	149	18.6
Exercise	13	1.6
Medication	166	20.7
*Western medication*	*156*	*19.5*
*Chinese traditional medicine*	*6*	*0.7*
*ICTWMI*	*4*	*0.5*
Health education	72	9.0
Psychological	21	2.6
Combination	381	47.5
*DE*	*34*	*4.2*
*DEM*	*130*	*16.2*
*DEMHP*	*217*	*27.1*

*ICTWMI* Integrated Chinese Traditional Medicine and Western Medicine Intervention

*DE intervention* Dietary + Exercise intervention

*DEM intervention* Dietary + Exercise + Medication intervention

*DEMHP intervention* Dietary + Exercise + Medication + Health education + Psychological intervention

### Quality of the studies

After analyzing the full texts of all 802 GDM health intervention articles from China based on the guideline of studies and the Cochrane Collaboration tool for assessing risk of bias, we found some of the articles were poor quality. While 39% (*n* = 316) failed to report the GDM diagnostic criteria used (Table 2), the remaining studies applied an assortment of 5 different international (*n* = 183, 22%) or 7 Chinese (*n* = 303, 38%) diagnostic standards. Moreover, although 639 (79%) of the 802 studies were randomized clinical trials, 396 (49%) articles failed to clearly describe the randomization methods.

**Table 2:** Information on the studies included in our analysis (*n* = 802)

Diagnostic criteria used to diagnose gestational diabetes mellitus	n (%)
Chinese criteria	303 (38%)
*Maternal Standard of Chinese Medical Association*	*87 (11%)*
*Guidelines for Pregnancy Associated with Diabetes*	*2 (2%)*
*Diabetic Standard of Chinese Medical Association*	*17 (2%)*
*Chinese Preventive Guidelines for Type 2 Diabetes*	*12 (1%)*
*New Chinese Traditional Medicine for Diabetes*	*1 (0.1%)*
*National Health Diagnostic Standard*	*2 (0.1%)*
*Clinical Diagnostic Criteria*	*170 (21%)*
International standards	183 (23%)
*World Health Organization*	*60 (7%)*
*American Diabetic Association*	*60 (7%)*
*The International Association of Diabetes and Pregnancy Study Groups*	*39 (5%)*
*National Diabetes Date Group*	*17 (2%)*
*Fernado*	*7 (0.8%)*
No diagnostic standards	316 (39%)
Study design	
Randomized controlled trials	639 (79%)
*Randomized method mentioned*	*243 (30%)*
*Randomized method not mentioned*	*396 (49%)*
Retrospective analysis	77 (10%)
Observation analysis	86 (11%)

### Health outcomes

Over 40 possible GDM health outcomes for mothers and newborn babies were identified, and of these, approximately 30 rarely occurred. The 7 most commonly reported adverse maternal health outcomes were: hypertensive disorders, preterm polyhydramnios, postpartum hemorrhage, premature rupture of membranes, ketoacidosis, postnatal infection and uterine-incision delivery, which occurred most frequently. The 7 most frequently reported adverse neonatal health outcomes were: macrosomia, neonatal hypoglycemia, neonatal hyperbilirubinemia, neonatal asphyxia, abnormal fetus, fetal death and fetal distress. Among those, macrosomia and neonatal hyperbilirubinemia were the most frequently-observed.

### Effectiveness of the interventions

Weighted pooled odds ratio meta-analyses were used to evaluate the effectiveness of different GDM treatment strategies on adverse maternal and infant health outcomes. Of the 802 studies reviewed, to ensure a certain level of study qualities, only the 278 meeting the following criteria were included in this analysis:1) measured maternal “C-section rate”, “pre-eclampsia”, “gestational hypertension” or “incidence of GDM”; 2) infant “birthweight”, “large or small-for-gestational age” or “macrosomia”; 3) study design was randomized, cases or placebo controlled; 4) the drop-out rate was less than 25%; 5) there were at least 10 subjects per group; 6) pregnant women ages 18–45 years old; 7) study duration was minimum 8 weeks, to ensure a treatment period from GDM diagnosis (approximately 28 weeks gestation) until delivery. The effect of individual GDM treatment strategies varied by outcomes: the lower the odds ratio, the most effective the GDM treatment on the outcome of interest, the closer to 1 the less effective (Table 3).

**Table 3:** Weighted pooled odds ratio meta-analyses on the effectiveness of GDM treatments (*n* = 278)

Complication	Odds ratio (95% CI)(N studies)										
	GMD Health interventions in China										
	Dietary	Exercise	Medication			Health education	Psychological	Combination			Overall
			Western medication	Chinese traditional medication	ICTWMI			DE	DEM	DEMHP	
Gestational hypertension	0.28***	0.53	0.39***	n/a	0.55	0.28**	0.33***	0.28 ***	0.24 ***	0.26***	0.29***
	(0.21, 0.37)	(0.14, 1.95)	(0.32, 0.49)		(0.12, 2.58)	(0.12, 0.67)	(0.19, 0.57)	(0.21, 0.38)	(0.16, 0.36)	(0.23, 0.31)	(0.27, 0.33)
	(24)	(1)	(41)		(1)	(4)	(5)	(14)	(11)	(67)	(168)
Polyhydramnios	0.2***	0.13	0.36***	0.24**	0.33*	0.21*	0.31**	0.23***	0.31***	0.23***	0.27***
	(0.13, 0.30)	(0.01, 1.16)	(0.29, 0.44)	(0.08, 0.70)	(0.110.99)	(0.04, 0.99)	(0.15, 0.65)	(0.16, 0.34)	(0.22, 0.43)	(0.19, 0.27)	(0.24, 0.30)
	(13)	(1)	(38)	(1)	(2)	(2)	(4)	(13)	(14)	(56)	(144)
Caesarean section	0.34***	0.67	0.49***	0.31***	n/a	0.10***	0.32	0.28***	0.36***	0.43***	0.39***
	(0.28, 0.41)	(0.34, 1.34)	(0.41, 0.58)	(0.17, 0.57)		(0.03, 0.38)	(0.08, 1.32)	(0.23, 0.35)	(0.24, 0.53)	(0.38, 0.48)	(0.37, 0.42)
	(18)	(2)	(33)	(2)		(1)	(1)	(16)	(8)	(54)	(135)
Fetal distress	0.27***	0.13	0.28***	0.28	0.22	n/a	0.54	0.29***	0.2***	0.24***	0.26***
	(0.19, 0.38)	(0.01, 1.16)	(0.16, 0.50)	(0.06, 1.42)	(0.02, 2.12)		(0.18, 1.65)	(0.19, 0.44)	(0.11, 0.34)	(0.20, 0.31)	(0.22, 0.30)
	(9)	(1)	(9)	(1)	(1)		(2)	(9)	(7)	(36)	(75)
Premature rupture of fetal membranes	0.27***	0.39	0.47*	n/a	n/a	0.27**	0.27**	0.24***	0.47***	0.33***	0.33***
	(0.18, 0.40)	(0.09, 1.61)	(0.26, 0.86)			(0.10, 0.72)	(0.11, 0.67)	(0.13, 0.43)	(0.30, 0.74)	(0.25, 0.42)	(0.28, 0.39)
	(6)	(1)	(7)			(2)	(2)	(5)	(7)	(26)	(56)
Preeclampsia	n/a	n/a	2.07	0.53	0.38	n/a	0.23	0.25***	0.10*	0.18***	0.34***
			(0.52, 8.31)	(0.27, 1.02)	(0.14, 1.03)		(0.02, 2.16)	(0.11, 0.58)	(0.01, 0.89)	(0.08, 0.40)	(0.24, 0.49)
			(1)	(2)	(1)		(1)	(3)	(1)	(4)	(13)
Macrosomia	0.23***	0.47	0.22***	0.38**	0.21**	0.16***	0.48*	0.20***	0.28***	0.25***	0.25***
	(0.19, 0.29)	(0.11, 1.99)	(0.17, 0.28)	(0.20, 0.76)	(0.07, 0.61)	(0.05, 0.47)	(0.24, 0.94)	(0.14, 0.30)	(0.20, 0.40)	(0.21, 0.30)	(0.22, 0.29)
	(27)	(1)	(35)	(2)	(1)	(3)	(3)	(12)	(12)	(74)	(170)
Premature delivery	0.25***	0.27*	0.75	0.46*	0.37	0.42	0.58	0.20***	0.24***	0.26***	0.28***
	(0.18, 0.34)	(0.08, 0.86)	(0.44, 1.25)	(0.25, 0.84)	(0.12, 1.20)	(0.18, 1.03)	(0.24, 1.36)	(0.14, 0.29)	(0.16, 0.37)	(0.22, 0.31)	(0.25, 0.31)
	(14)	(2)	(7)	(2)	(1)	(2)	(3)	(13)	(9)	(54)	(107)
Asphyxia neonatorum	0.29***	0.47	0.29***	0.27**	0.18*	0.65	n/a	0.24***	0.19***	0.20***	0.24***
	(0.20, 0.44)	(0.11, 1.99)	(0.18, 0.45)	(0.11, 0.65)	(0.04, 0.89)	(0.11, 4.08)		(0.10, 0.55)	(0.12, 0.31)	(0.15, 0.27)	(0.20, 0.28)
	(10)	(1)	(12)	(2)	(1)	(1)		(4)	(9)	(33)	(73)
Hypoglycemia	0.18***	0.47	0.18***	n/a	n/a	0.20*	0.12	0.12***	0.27***	0.25***	0.20***
	(0.11, 0.31)	(0.11, 1.99)	(0.13, 0.27)			(0.05, 0.73)	(0.01, 1.04)	(0.08, 0.19)	(0.16, 0.45)	(0.19, 0.32)	(0.17, 0.24)
	(8)	(1)	(19)			(2)	(1)	(8)	(6)	(23)	(68)
Hyperbilirubinemia	0.54	n/a	0.17***	n/a	0.28	n/a	n/a	0.22***	0.20***	0.31***	0.23***
	(0.19, 1.52)		(0.13, 0.22)		(0.05, 1.53)			(0.13, 0.39)	(0.13, 0.30)	(0.25, 0.40)	(0.20, 0.27)
	(2)		(29)		(1)			(4)	(8)	(21)	(65)

*P ≤ 0.05; **P ≤ 0.01; ***P ≤ 0.001

Table 3 showed weighted pooled odds ratio meta-analyses on the effectiveness of GDM treatments. Among all the GDM health intervention strategies employed in China, dietary, Western medication, and combined interventions were the most effective in reducing the probabilities of maternal and infant adverse outcomes. Combined interventions were highly effective (overall *p* value <0.001 for all interventions) in reducing all adverse health outcomes. Dietary and Western medication were generally effecitive, although dietary intervention did not have a statistically significant effect on preeclampsia and Western medication did not improve the odds of hyperbilirubinemia and premature delivery. Exercise, Chinese traditional medication, health education and psychological interventions were the least effective, and rarely worked when not integrated with other interventions. However, since they were employed in few studies, their small sample sizes may have biased the meta-analysis results.Forest plots of GDM treatment effectiveness were omitted due to the limited space, but provided in Additional file 1.

## Discussion

Social changes combined with decades of economic reform in China have resulted in a transition from the traditional Chinese lifestyle and dietary patterns being replaced by with “Western” lifestyle habits and dietary patterns. These changes have had a dramatic impact on the demographics of China’s health and diseases. Over the past 20 years, China has seen a sharp rise in obesity and related non-communicable diseases, including type II diabetes mellitus and GDM. Aside from the increased health risks to the mother, the offspring born from mothers with GDM are more prone to obesity and related metabolic disorders such as cardiovascular diseases in later life [27]. Therefore, the detection and treatment of GDM is necessary to identify women at risk of short- and long-term complications, and ameliorate the health outcomes in the offspring.

Thus far, several systematic reviews have been published on various interventions, either single [28] or different interventions [29], for the management of GDM in other countries, and only a few have evaluated preventive intervention programs [30–32]. The goal of this study was to review the most widely-used GDM healthcare interventions in China and to the best of our knowledge, this is the first study to do so. We selected 802 published articles that reported the outcomes of independent studies evaluating various healthcare treatment interventions in pregnant women with GDM. The first GDM healthcare intervention study in China was published in 1997; from that time onwards, the number of published articles on GDM healthcare interventions in China rapidly increased. This not only reflects the rising incidence of GDM in China over the last 20 years, but it also highlights a growing awareness among researchers and healthcare practitioners on the importance of addressing this problem.

In our analysis, indeed, we observed that a wide variety of intervention strategies used to address GDM in China. Almost half of the reviewed studies reported combination interventions that combined different types of interventions with the goal of managing GDM [33]. Among all the possible combination interventions, dietary + exercise + medication + health education + psychological (DEMHP) interventions accounted for highest percentage (27.4%) of these studies. The initial treatment interventions with the greatest number of studies was found to be either diet alone or diet in combination with another intervention. It is estimated that the majority of women with GDM can achieve target glycemic control through diet and exercise (DE) [34, 35] and this is consistent with the recommendations by American Diabetic Association, according to which all pregnant women with GDM need an individual dietary plan tailored to the individual’s health status (including height and weight) [36, 37]. However, DE interventions are less common in China compared with other Western countries, highlighting a need to gain a better understanding of the comparative and cost-effectiveness of such programs in China [38]. When dietary interventions were shown to be ineffective at controlling GDM, medication was added to the treatment regimen. Consistent with professional guidelines insulin was the most frequently-used medication [39, 40]. Surprisingly, only a few studies examined the use of traditional Chinese medicines [41, 42] or the integration of Chinese traditional medicine and Western medicine. Given the long history of the use of traditional Chinese medicine and concomitant Chinese therapies, a better understanding is needed of the efficacy of these therapies and their roles as part of an integrated healthcare solutions in China. The least frequent approach to the control of GDM was found to be with the use of some form of psychological interventions [43]. Finally, a few studies employed combined DEMHP interventions to control GDM in early stages in China, which showed very high positive effects [44, 45].

The results obtained suggest that the interventions studied may be effective in reducing the risk of several adverse maternal and short-term neonatal outcomes in China. However care needs to be taken in interpreting these results. The observed protective effects could have been overestimated considering the low quality of the evidence included in this analysis. Nevertheless, scientific findings from other countries indicates that even if glycaemic control is achieved through lifestyle or therapy interventions, there is a clear impact on a limited number of maternal and offspring short term outcomes. Treatment of GDM has been found to significantly reduce the risk of pre-eclampsia and macrosomia [46]. While dietary interventions for women with GDM, have been studied for their efficacy against a variety of short and long-term maternal and offspring outcomes, and have only been associated with less frequent insulin use and a lower birthweight [47], or with a reduction in caesarean sections [30]. In contrast, the current study identified a considerable effect of dietary interventions on the majority of maternal and infant health outcomes in China.

This study also evaluate the effects of pharmacological interventions. Evidence supports the use of oral antidiabetic drugs in China, particularly metformin alone or in combination with insulin to reduce the risk of adverse maternal and neonatal outcomes, including pregnancy induced hypertension, neonatal hypo glycemia, and the need for NICU admission [48]. Our data showed that the combination of metformin and insulin performed better than insulin alone. To date no evidence exists for evidence-based psychological interventions, and neither do guidelines addressing the psychosocial management of GDM patients [49]. In this review, the studies incorporating this dimension did not show a greater effects than the lifestyle or pharmacological interventions.

The first limitation of this study was that all studies were included in the review without regards to the criteria used for randomizing participants, study methodology, treatment dose, or duration. For these reasons, it is likely that some low-quality studies were included in the full text extractions and data analysis. Second, we did not perform any power analyses nor statistical analyses to evaluate the effectiveness of interventions, as this was out of the scope: most of the identified studies were small and heterogeneous, especially in regards to maternal age, weight status, risk for GDM and treatment periods. Thus, a comparison of the effectiveness of the different types of GDM treatment interventions is challenging and all comparisons should be reviewed with caution. Third, the over-restrictive inclusion criteria may have limited our ability to identify GDM prevention studies. Four, the majority of the studies were based in urban areas in Eastern China; further data would be needed from rural China to have a more complete national overview. Fifth, the meta-analysis may be subject to publication bias with only studies with positive finding published and may not necessarily reflect the true practice in China. Finally, we only considered published original research articles, excluding reports, agency work, dossiers, and other types of documents that may also be used by clinicians and decision makers.

## Conclusion

Our study is a synthesis of the existing healthcare interventions to control GDM in China and shows objective data supporting an increasing interest in GDM and research efforts to identify the best approach to control this condition. The growing prevalence of GDM and its associated adverse maternal and neonatal outcomes among China’s huge population suggests a substantial economic cost; more data are urgently needed to inform national healthcare policies in order to facilitate the prevention and treatment of GDM. Further research and clinical evidence is particularly needed with regards to GDM prevention programs in China which appear to be missing and may not be implemented at a national level. Although lots of treatment interventions have been employed in China, the most important item is create a good environment to implement these treatment strategies [50]. Although we identified 6 major types of interventions in China, we failed to find interventions utilizing digital technology or cellphones to monitor glucose levels and implementations of GDM treatments. Well-designed clinical trials are also needed to determine the most effective control and prevention treatments, incorporating cost effectiveness evaluations.

## Additional file

Additional file 1: Forest Plots. (DOCX 6725 kb)

## Abbreviations

CNKI: China National Knowledge Infrastructure
DE: Dietary + Exercise
DEM: Dietary + Exercise + Medication
DEMHP: Dietary + Exercise + Medication + Health education + Psychological
GDM: Gestational Diabetes Mellitus
ICTWMI: Integrated Chinese Traditional Medicine and Western Medicine
