# Supplementary material for: Healthcare interventions for the prevention and control of gestational diabetes mellitus in China: a scoping review
Source: BMC Pregnancy Childbirth. 2017 Jun 5;17:171. doi: 10.1186/s12884-017-1353-1 (PMC5460547; doi:10.1186/s12884-017-1353-1)

**Appendix 1 Forest plot：Treatment for gestational diabetes mellitus on gestational hypertension**


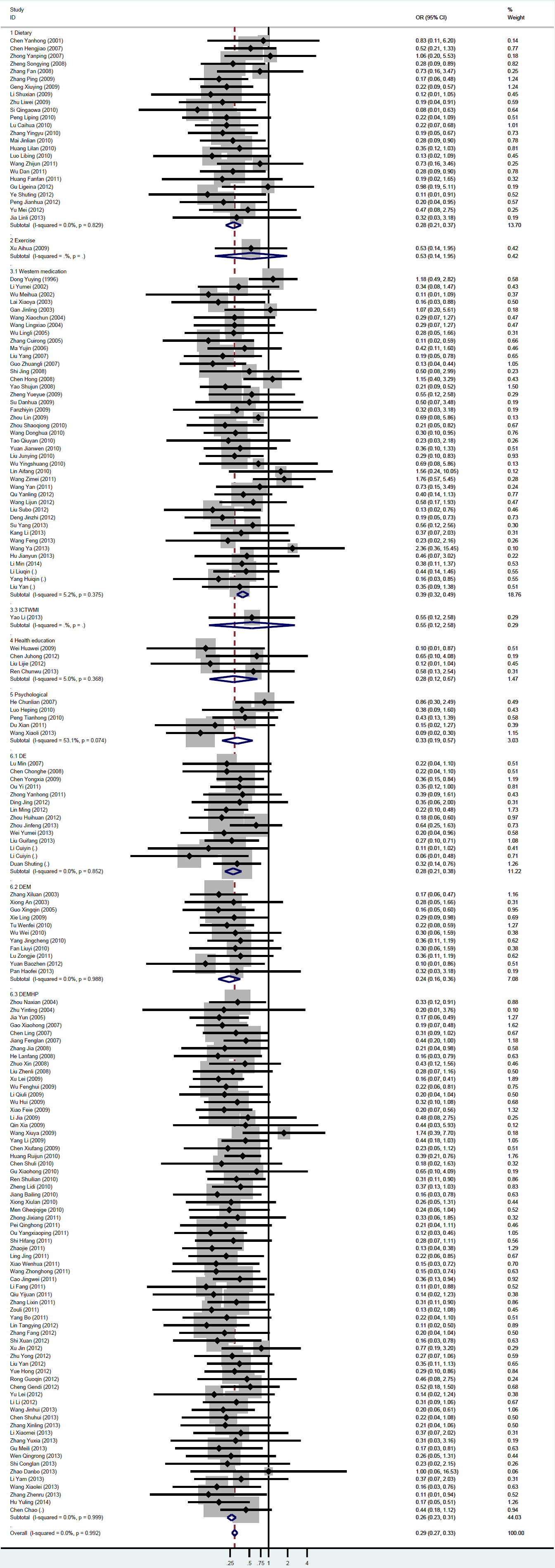


**Appendix 2 Forest plot：Treatment for gestational diabetes mellitus on polyhydramnios**


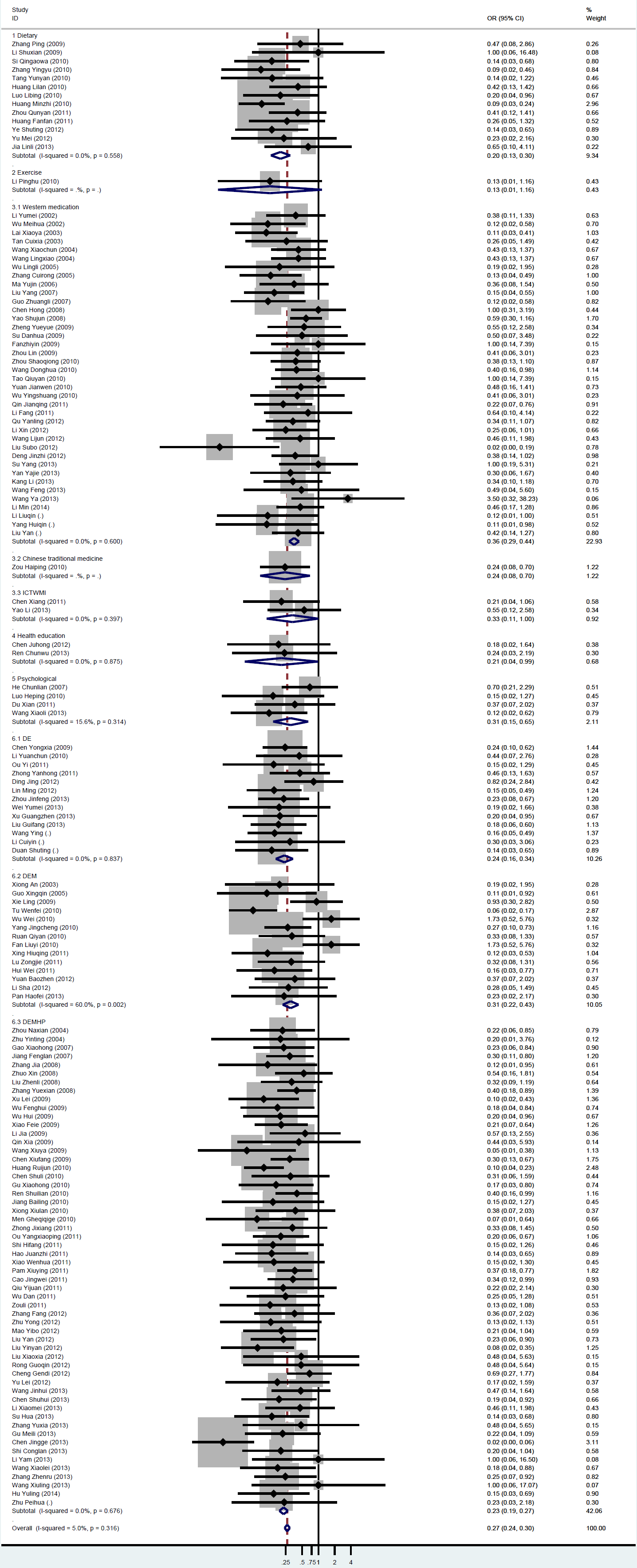


**Appendix 3 Forest plot：Treatment for gestational diabetes mellitus on Caesarean section**


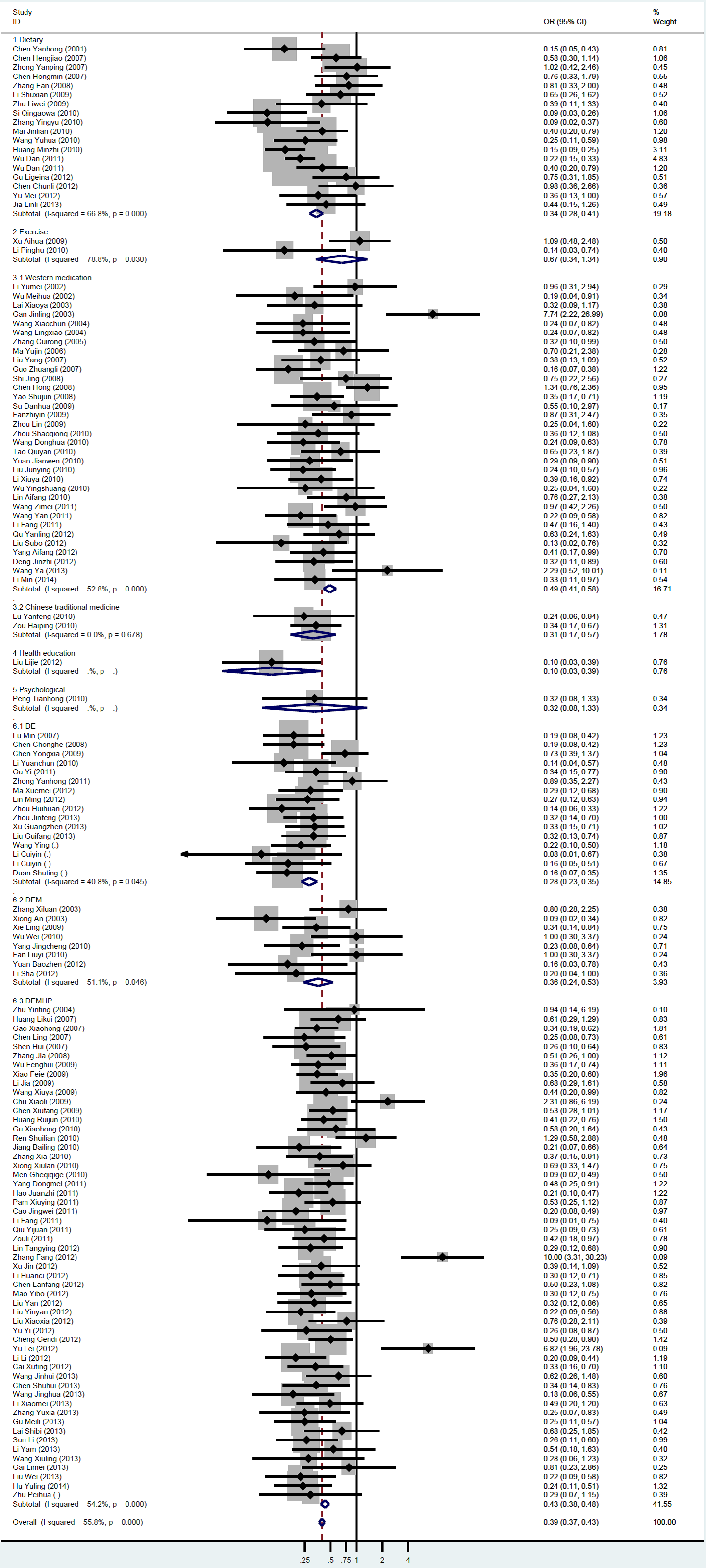


**Appendix 4 Forest plot：Treatment for gestational diabetes mellitus on fetal distress**


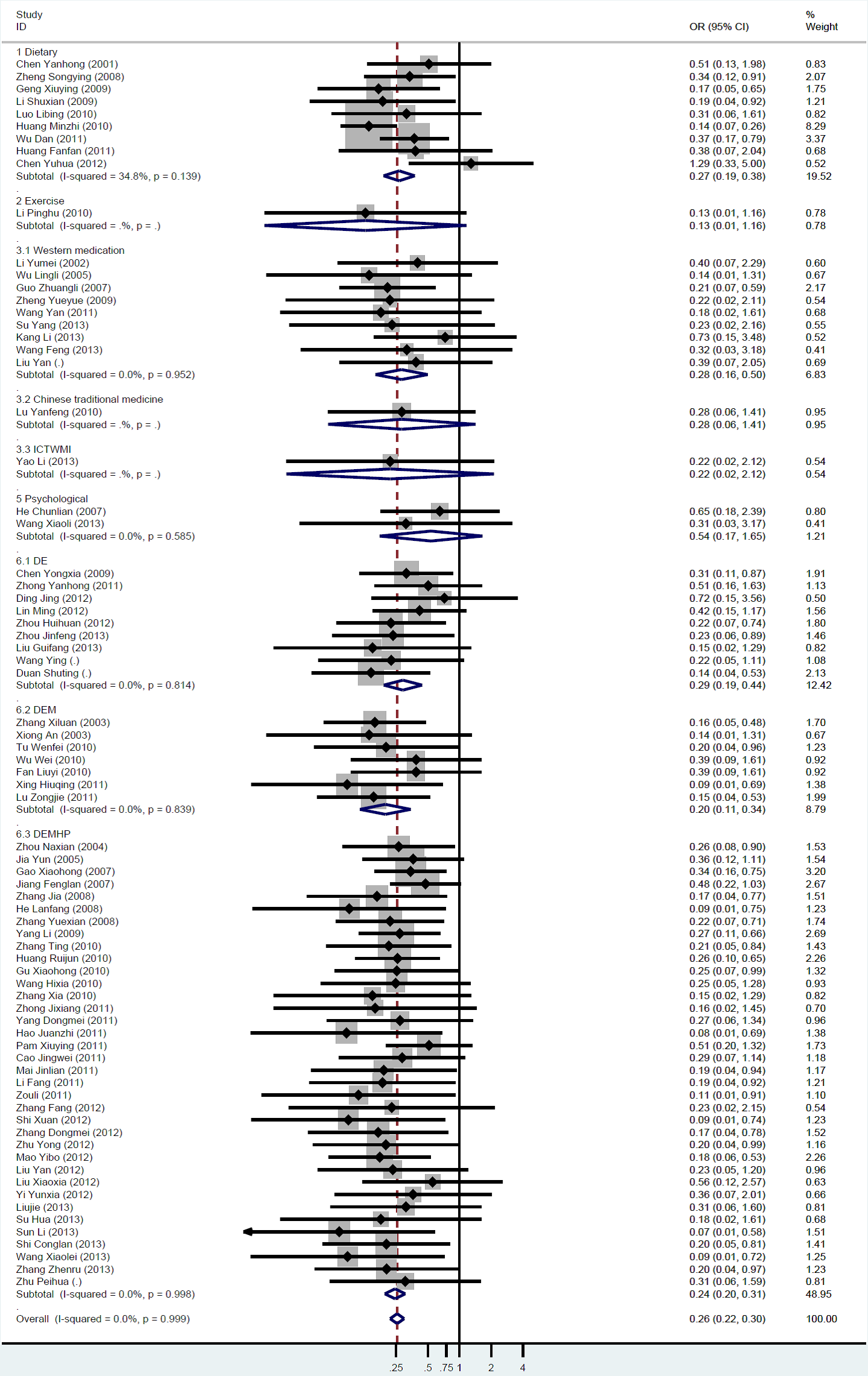


**Appendix 5 Forest plot：Treatment for gestational diabetes mellitus on premature rupture**


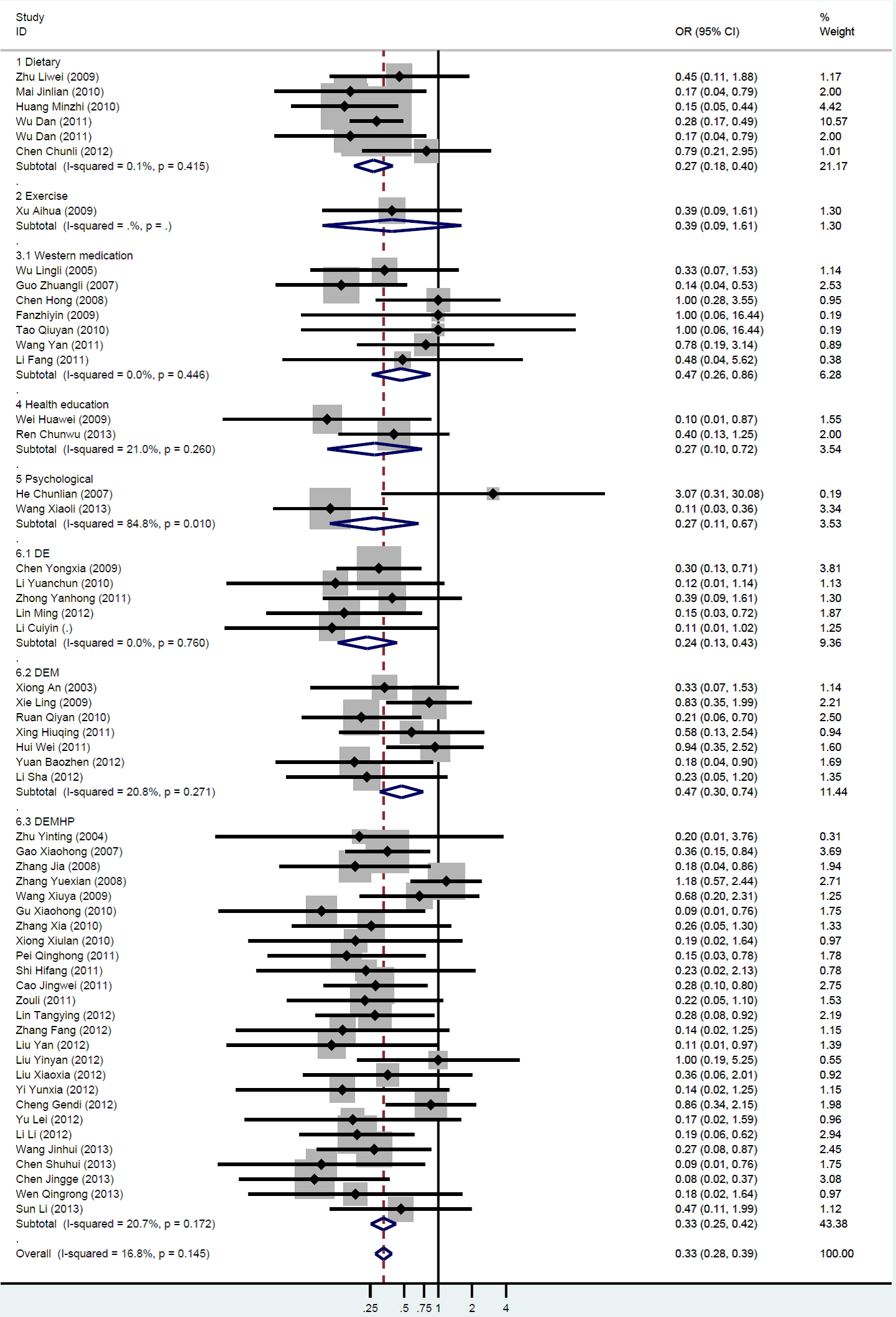


**Appendix 6 Forest plot：Treatment for gestational diabetes mellitus on preeclampsia**


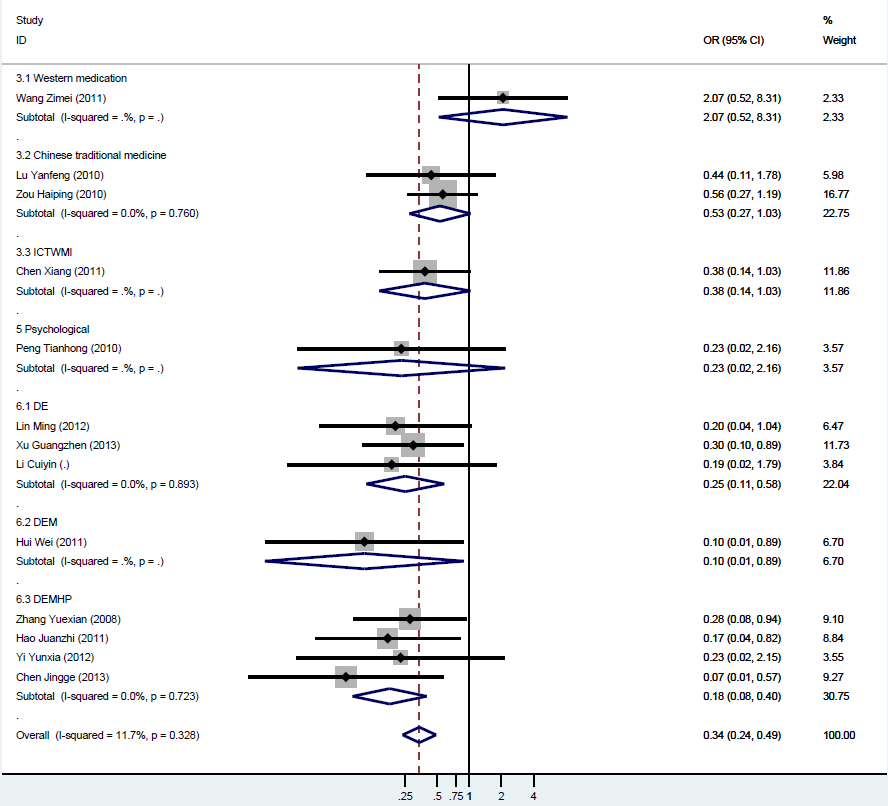


**Appendix 7 Forest plot：Treatment for gestational diabetes mellitus on macrosomia**


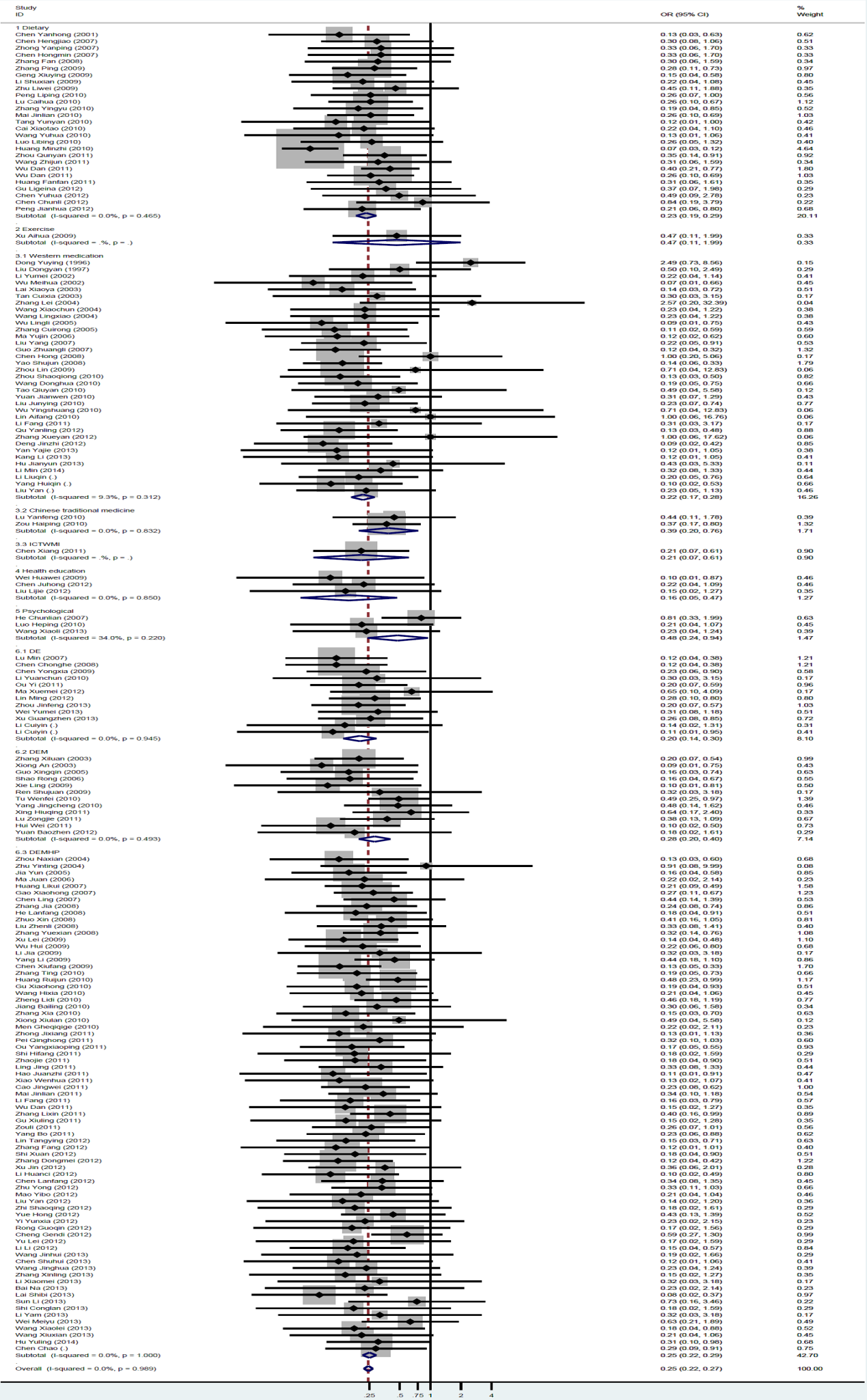


**Appendix 8 Forest plot：Treatment for gestational diabetes mellitus on premature delivery**


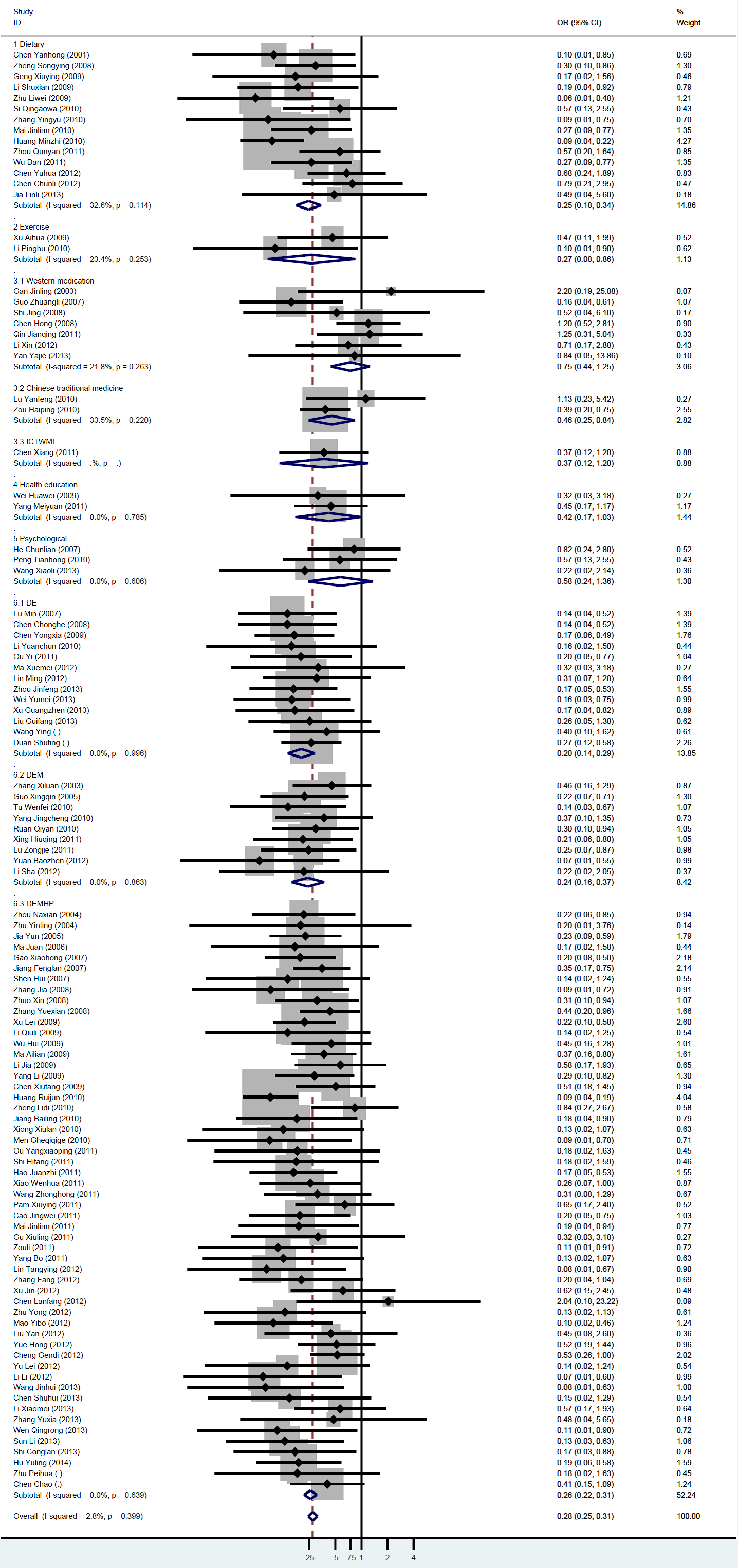


**Appendix 9 Forest plot：Treatment for gestational diabetes mellitus on asphyxia neonatorum**


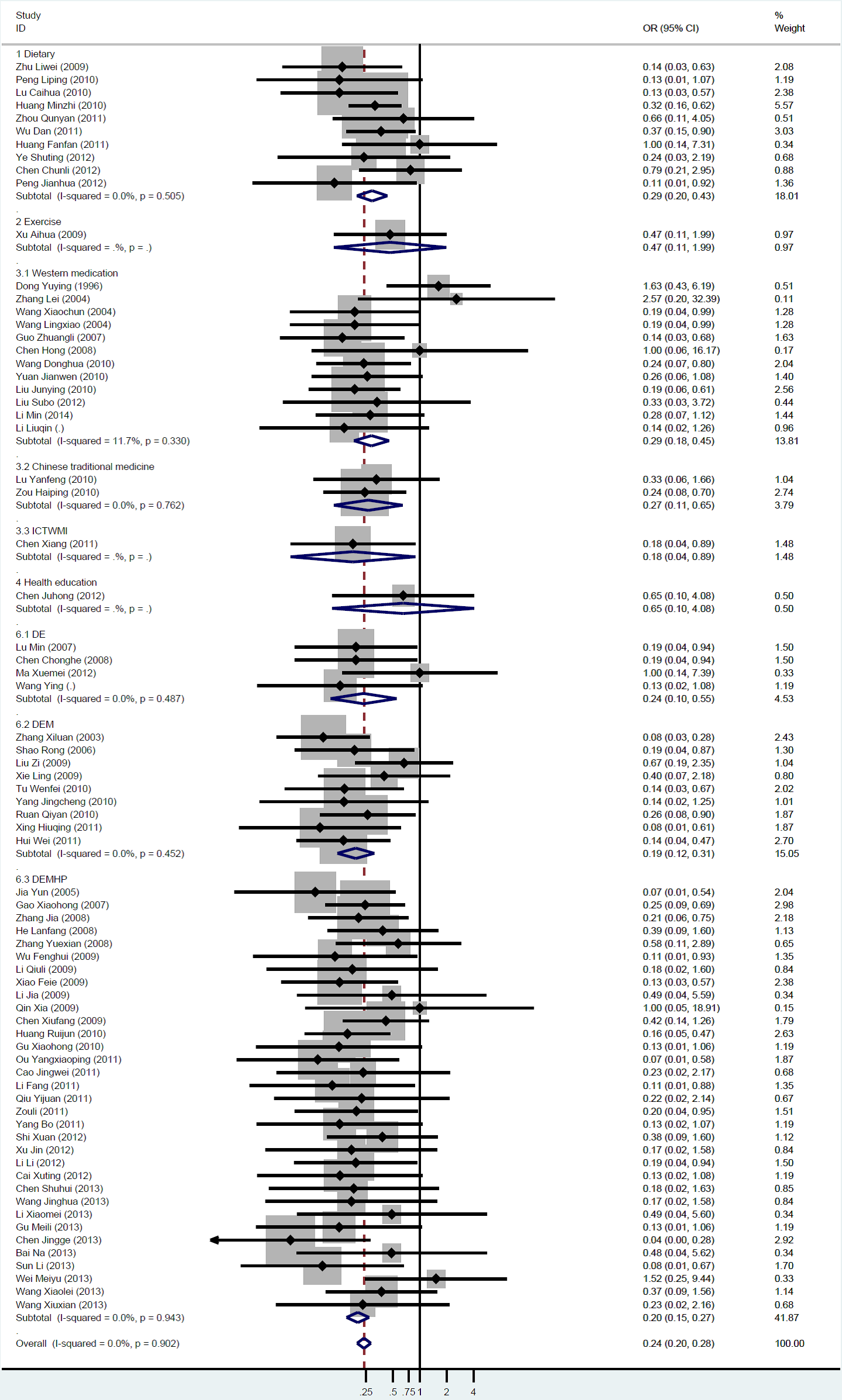


**Appendix 10 Forest plot：Treatment for gestational diabetes mellitus on hypoglycemia**


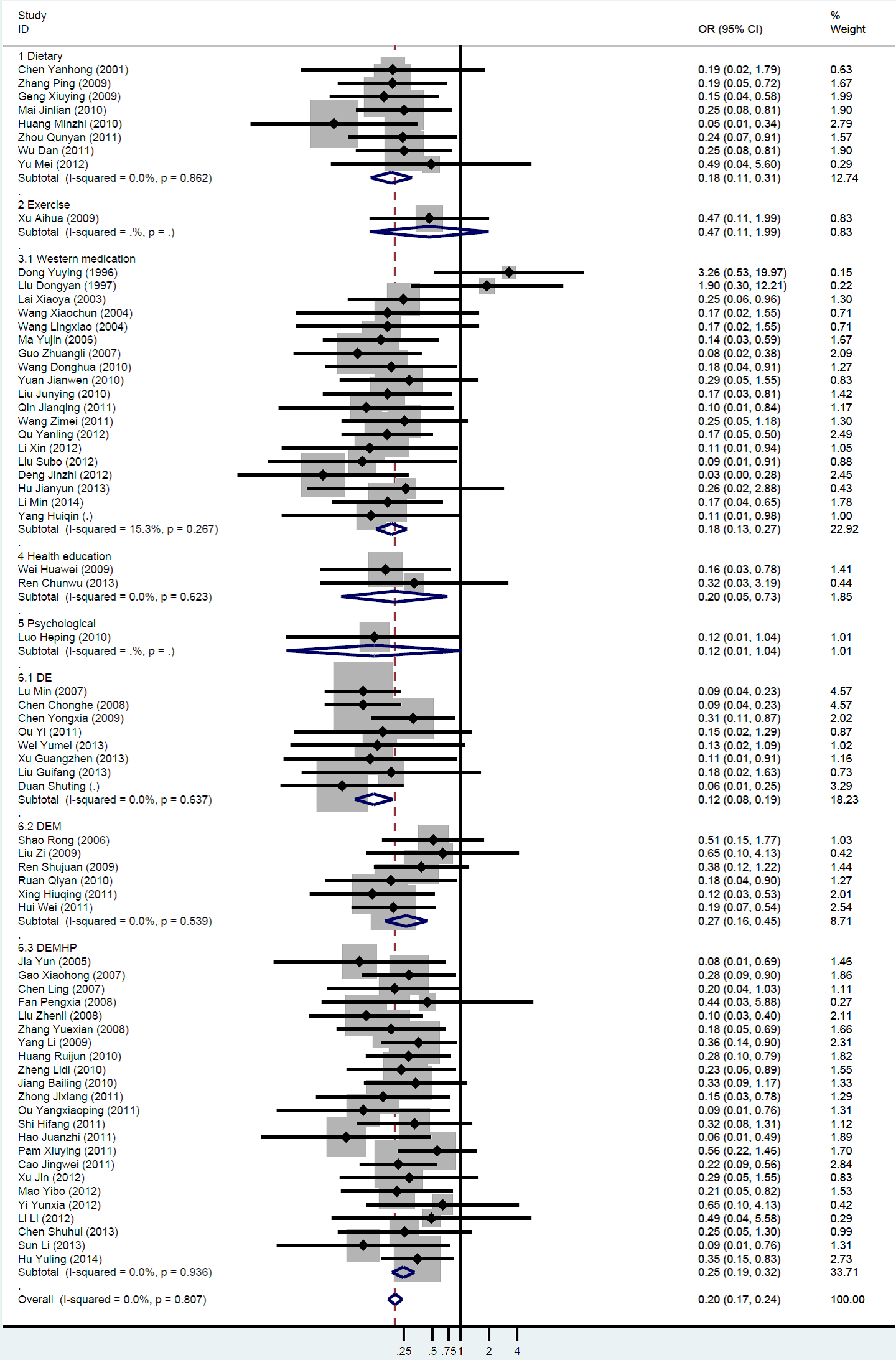


**Appendix 11 Forest plot：Treatment for gestational diabetes mellitus on hyperbilirubinemia**


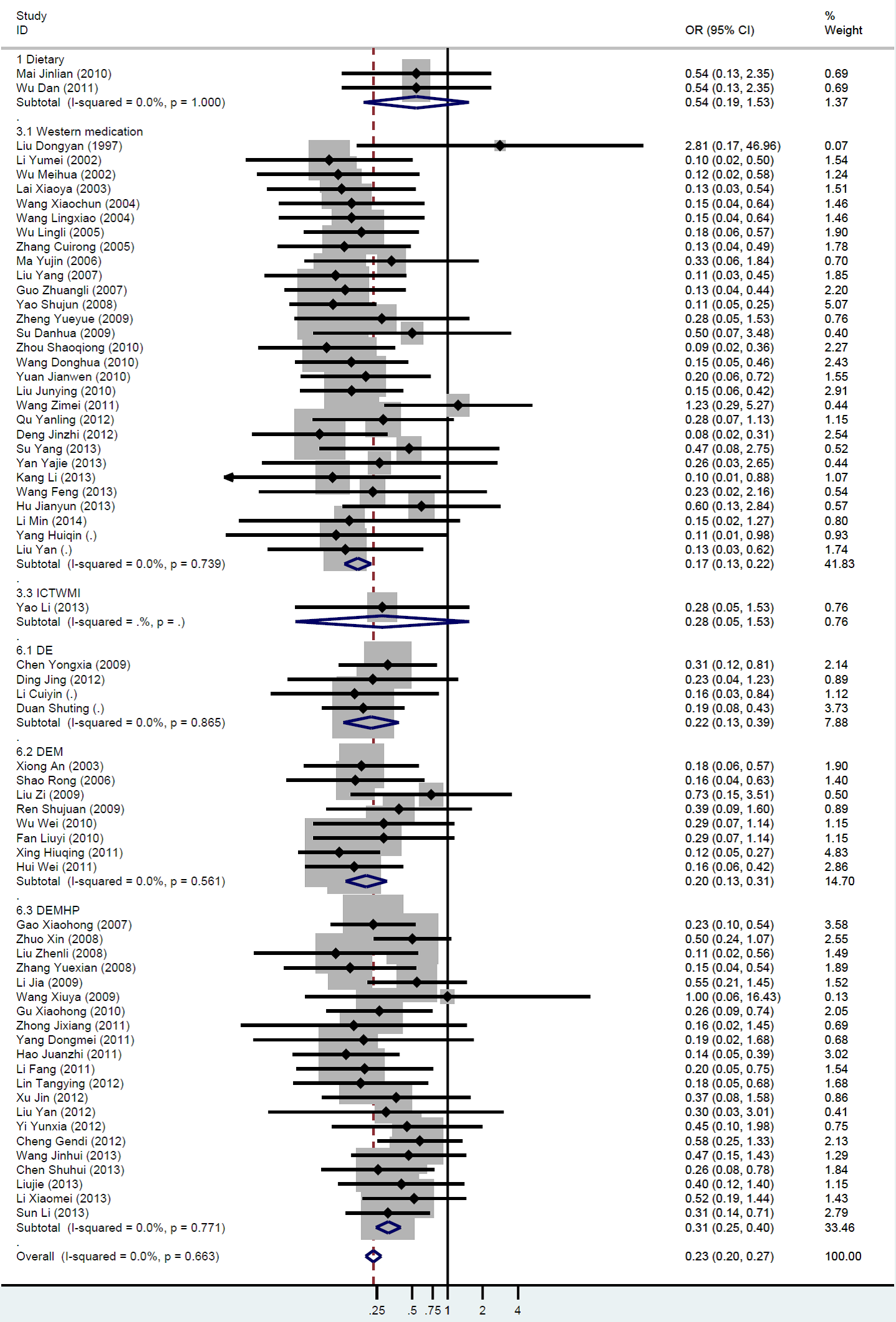

Supplement: Additional file 1: — Forest Plots. (DOCX 6725 kb) [file 12884_2017_1353_MOESM1_ESM.docx]
